# Bayesian Species Delimitation Can Be Robust to Guide-Tree Inference Errors

**DOI:** 10.1093/sysbio/syu052

**Published:** 2014-08-05

**Authors:** Chi Zhang, Bruce Rannala, Ziheng Yang

**Affiliations:** ^1^Department of Bioinformatics and Genetics, Swedish Museum of Natural History, Box 50007, SE-104 05 Stockholm, Sweden; ^2^Genome Center and Section of Evolution and Ecology, University of California at Davis, One Shields Avenue, Davis, CA 95616, USA; ^3^Department of Genetics, Evolution and Environment, University College London, Darwin Building, Gower Street, London WC1E 6BT, UK; and ^4^Center for Computational Genomics, Beijing Institute of Genomics, Chinese Academy of Sciences, Beijing 100101, China

Species limits are traditionally determined based on morphological, behavioral, and ecological traits. In recent years, genetic sequence data have increasingly been used to delimit species due to the advancement of sequencing technologies and development of statistical methods of data analysis (Wiens 2007; Fujita et al. 2012). Early methods relied on reciprocal monophyly in the reconstructed gene trees, fixed sequence differences between putative species, or simple cut-offs on migration rates or genetic distances between putative species (Sites and Marshall 2004). More recent methods are based on the multispecies coalescent model (Rannala and Yang 2003) and avoid arbitrary cut-offs (Knowles and Carstens 2007). Among the recent methods, the Bayesian method of Yang and Rannala (2010) has a number of advantages over its competitors (Fujita and Leaché 2011). The Bayesian method uses Bayesian model selection to compare different species-delimitation models in the multispecies coalescent framework, and uses reversible-jump Markov chain Monte Carlo (rjMCMC) to estimate the posterior probabilities for different delimitation models. The method accommodates multiple loci, and does not require reciprocal monophyly of inferred gene trees. The underlying multispecies coalescent model accounts for incomplete lineage sorting and species-tree–gene tree conflicts due to ancestral polymorphism. The likelihood calculation on sequence alignments allows the method to make a full use of the information in the data while accounting for the uncertainties in the gene tree topologies and branch lengths. Compared with traditional morphology-based taxonomic practice, which varies widely across taxonomic groups, the Bayesian method infers species status from a genealogical and population genetic perspective and is arguably more objective (Fujita and Leaché 2011; Fujita et al. 2012).

In computer simulations, the Bayesian method was found to have good statistical properties (Leaché and Fujita 2010; Zhang et al. 2011; Camargo et al. 2012), with low false positives (the error of splitting one species into two) and false negatives (the error of failing to recognize distinct species). Simulations also suggest that the method has good power in identifying distinct species in the presence of small amounts of gene flow, and is not misled to infer geographical populations as distinct species when the migration rate is high (Zhang et al. 2011).

To reduce the space of models to be evaluated in the rjMCMC, the implementation of (Yang and Rannala 2010; Rannala and Yang 2013) in the program bpp (for Bayesian Phylogenetics and Phylogeography) requires the user to specify a rooted phylogeny for the populations, called the guide tree. The program then evaluates only those models that can be generated by collapsing nodes on the guide tree. The program currently does not change the relationships among the populations, nor does it split a population into different species.

As a simple evaluation of the impact of the guide tree on species delimitation by bpp, Leaché and Fujita (2010) randomized the populations at the tips of a 10-population guide tree for West African forest geckos and found that the incorrect guide tree caused bpp to over-split. When closely related populations that belong to the same species are incorrectly separated on the guide tree and are grouped with more distant populations, bpp tends to infer all of them as distinct species. However, the analysis of Leaché and Fujita (2010) is on a small scale, and furthermore, the random guide trees generated by permutation may be too wrong, unlikely to be encountered in real data analysis when the guide tree is estimated from real data. Here, we conduct a simulation study to examine the performance of the method under more realistic scenarios, that is, when the guide tree is inferred from the sequence data.

A number of heuristic methods have been used to construct the guide tree, including:

a) clustering algorithms such as structure (Pritchard et al. 2000; Falush et al. 2003), structurama (Huelsenbeck and Andolfatto 2007), or baps (Corander et al. 2004), which can assign individuals to populations and even infer a population tree. Those methods are often applied to microsatellite data or single-nucleotide polymorphisms (SNPs).
b) phylogenetic methods such as RAxML (Stamatakis 2006) and MrBayes (Ronquist et al. 2012) applied to either a mitochondrial locus or concatenated nuclear loci.
c) species-tree methods such as best (Liu 2008) or *beast (Heled and Drummond 2010) applied to multiple nuclear loci.
d) species-discovery methods such as that of O'Meara (2010).
e) empirical population phylogeny based on geographical distributions or morphological and ecological characters.

A useful review of strategies for generating the guide tree used in recent studies of species delimitation by bpp has been provided by Carstens et al. (2013, table 1). Geographical distributions and morphological and ecological features of the populations are always important to defining putative species. However, it is difficult to consider such information in a simulation. In this study, we examine strategies b and c for obtaining a guide tree by analyzing DNA/RNA sequence data. The first approach we examine (strategy b) uses phylogenetic analysis of a mitochondrial locus. Note that in vertebrates, the mitochondrial genome has a much higher mutation rate than the nuclear genome so that the sequence data are more variable and more informative (e.g., Zhou et al. 2012). Furthermore, the effective population size for a mitochondrial locus is only one-fourth that for a nuclear locus, so that incomplete lineage sorting is less likely to occur and the mitochondrial gene tree is more likely to match the species/population phylogeny. This method has been used by Leaché and Fujita (2010), Hamback et al. (2013), Linde et al. (2014), among others. We use the program RAxML (Stamatakis 2006) to infer the unrooted maximum-likelihood (ML) tree and mid-point rooting to generate the rooted tree to be used as the guide tree for bpp. The program is widely used and provides a fast method to infer gene trees using ML. We also used the Bayesian method to infer rooted gene trees for the mitochondrial locus under the molecular clock, using the program beast (Drummond and Rambaut 2007), but we expect the results to be similar to the ML method.

**Table 1.** Parameter values used in simulating sequences at the nuclear loci

Species tree	τs	θs
Low-mutation rate		
Tree 1	(0.001, 0.002, 0.003)	0.002
Tree 2	(0.001, 0.001, 0.003)	0.002
High-mutation rate		
Tree 1	(0.01, 0.02, 0.03)	0.02
Tree 2	(0.01, 0.01, 0.03)	0.02

Notes: At the mitochondrial locus, the mutation rate is assumed to be 20 times as high as at a nuclear locus, and the population size is assumed to be one-fourth as large. Thus, $\tau_{\text{mt}}=20\tau_{\text{nuc}}$ and $\theta_{\text{mt}}=5\theta_{\text{nuc}}$.

The second approach we examine (strategy c) is use of species-tree methods applied to multiple nuclear loci. We use *beast (Heled and Drummond 2010) for this purpose. We note that it is possible to apply a traditional phylogenetic method such as ML to the concatenated nuclear data, but concatenation is in general inferior to species-tree methods based on the multispecies coalescent model (see Degnan and Rosenberg [2009] and Edwards [2009] for reviews). The strategy of using *beast to infer the guide tree for species delimitation by bpp has been used by Leaché and Fujita (2010), Linde et al. (2014), Satler et al. (2013), among others.

To keep the complexity of our simulation manageable, we do not consider the problem of assignment errors in this study and assume that the individuals are correctly assigned to the populations (see discussions later).

## Simulation Design

### Simulation of Sequence Data

**Figure 1.**
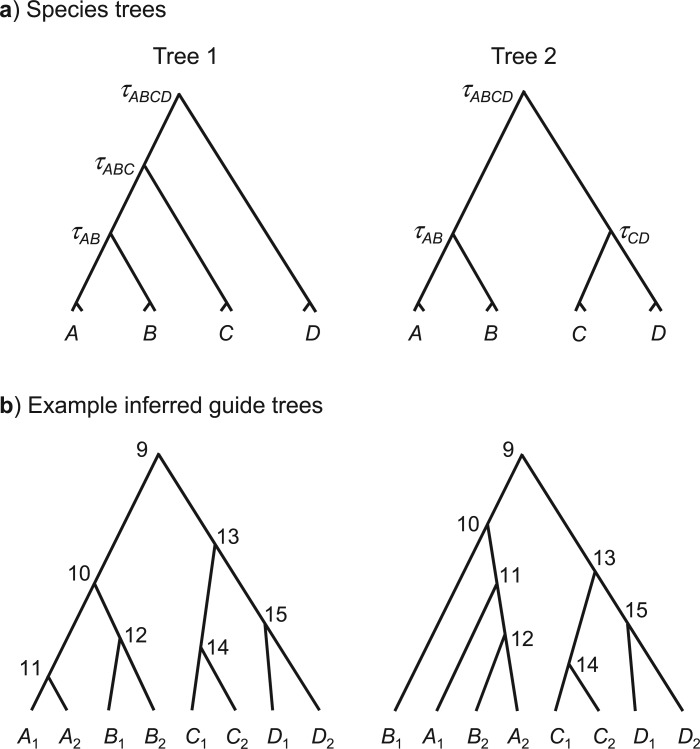
a) Two true species trees used for simulating sequence data under the multispecies coalescent model. Parameters in the model include the three species divergence times (τs) and the population size parameters (θs) for the seven species. Each species is split into two populations in the species-delimitation analysis by bpp, with eight populations on the guide tree. Either three or five sequences are sampled from each populations, with either 24 or 40 sequences in the alignment at each locus. b) Two inferred population (guide) trees, with interior nodes labeled.

We used two species trees, each of four species, to simulate the sequence data under the multispecies coalescent model (Rannala and Yang 2003). Tree 1 is balanced while tree 2 is unbalanced (Fig. 1). Parameters in the model include three species divergence times (τs) as well as seven population size parameters (θs) for the extant and extinct species on the species tree. Both τ and θ are measured by the expected number of mutations per site. For example, $\tau_{\text{AB}}=0.01$ in species tree 1 of Figure 1a means that the average sequence divergence from the time of the AB ancestor to the present is 1%, whereas θ=0.02 means that two random sequences sampled from the same population are 2% different on average. We assumed that each of the four species (*A*–*D*) consisted of two populations (labeled $A_{\text{1}},A_{\text{2}},B_{\text{1}},B_{\text{2}}$, etc.), so that there are eight populations on the guide tree. We consider two sample sizes, with three or five sequences sampled at each locus from each of the eight populations (i.e., with 24 or 40 sequences in the alignment for each locus). The program mccoal in bpp version 2.1c was used to generate gene trees with coalescent times (branch lengths) under the multispecies coalescent model (Rannala and Yang 2003) and to simulate sequence alignments given the gene trees. For each species tree, two sets of parameters were used, mimicking two different mutation rates (Table 1). We simulated either one or five nuclear loci as well as one mitochondrial locus. We assumed that the mutation rate at the mitochondrial locus was 20 times as high as at a nuclear locus and that the population size was one-fourth that for a nuclear locus so that $\tau_{\text{mt}}=20\tau_{\text{nuc}}$ and $\theta_{\text{mt}}=5\theta_{\text{nuc}}$ (e.g., Zhou et al. 2012). The JC69 mutation model (Jukes and Cantor 1969) was assumed both to generate and to analyze the sequence alignments. Note that the role of the mutation model here is to correct for multiple hits to estimate the gene tree topology and branch lengths, and that JC69 is deemed adequate for analysis of such highly similar sequences (Burgess and Yang 2008); in previous studies, even the infinite sites model produced very similar results (Satta et al. 2004).

The mutation rate was assumed to be constant over lineages (i.e., the molecular clock holds), and across the nuclear loci. The sequence length is 1000 sites for the mitochondrial locus and 500 sites for each nuclear locus. Sequences from the same species are assumed to coalesce freely (i.e., there is random mating even between individuals from different populations of the same species). We therefore simulated either 6 or 10 sequences from each of the four species and then randomly partitioned them into two populations of 3 or 5 sequences each.

### Generation of the Guide Tree from Phylogenetic Analysis Applied to a Mitochondrial Locus(*RAxML*)

The gene tree was inferred by a ML analysis of the mitochondrial locus using RAxML version 7.4.2. The nuclear loci were not used. The correct population assignment of sequences was assumed. The three or five sequences from each of the eight populations were constrained to be monophyletic. The substitution model used was GTR, since RAxML does not implement the JC69 model. Also, RAxML does not implement the molecular clock and infers unrooted trees instead. We used mid-point rooting to generate a rooted tree, which was used as the guide tree in later bpp analysis. As an alternative phylogenetic method, we also used the Bayesian method implemented in the program beast (Drummond and Rambaut 2007) to infer a rooted gene tree under the molecular clock, to be used as the guide tree in the bpp analysis.

### Generation of the Guide Tree using a Species-Tree Estimation Method Applied to the Nuclear Loci (*beast)

The species tree was inferred from the simulated nuclear loci using *beast in beast2 (version 2.0.2). Only the nuclear loci were analyzed, and the mitochondrial locus was not used in the *beast analysis. Again the correct population assignment of sequences was provided to the program. In other words, the three or five sequences for each of the eight populations ($A_{\text{1}},A_{\text{2}},B_{\text{1}},B_{\text{2}},C_{\text{1}},C_{\text{2}},D_{\text{1}}$, and $D_{\text{2}})$ were assigned to the same population, whereas the program estimates the phylogenetic relationships among the eight populations. Note that this is not the same as constraining the three sequences from the same population to be monophyletic on the gene tree. The multispecies coalescent model, while placing constraints on the gene tree, allows non-monophyly of sequences from the same species (see, e.g., figure 1 in Rannala and Yang [2003]). We followed the common practice and used the default improper priors in *beast, but note that proper priors may be preferable in real-data analysis. The prior on node ages was specified using a Yule process with an improper prior on the birth rate f(λ)=1/λ, 0<λ<∞. The population size parameters (θs) were assigned improper priors f(θ)=1/θ,0<θ<∞. The mutation model used was JC69, as in the simulation. The mutation rate was set to 1 so that time is measured by the number of mutations per site. A total of 3000 species trees were collected from the MCMC algorithm by sampling every 2000 iterations ($6\times 10^{\text{6}}$ iterations in total). The last 2200 trees were used to generate the maximum *a posteriori* (MAP) tree, to be used as the guide tree in the bpp analysis. In pilot runs, the same analysis was conducted twice to confirm consistency between runs.

Note that as we assume the correct assignment, the only errors that *beast and RAxML can make will concern the relationships among the eight populations. Figure 1b shows two possible inferred population (guide) trees. The tree on the left is correct under species tree 2, but the one on the right is wrong regardless of whether species tree 1 or tree 2 is the true tree.

### bpp Analysis

The guide tree was either the ML tree for the mitochondrial locus inferred by RAxML or the MAP tree inferred from the nuclear loci by *beast, as described above. Given the guide tree, the nuclear sequence data (either one locus or five loci) simulated above were analyzed using bpp version 2.2 to delimit species. The mitochondrial locus was not used in the bpp analysis.

The divergence time for the root of the guide tree ($\tau_{\text{ABCD}}$ or $\tau_{\text{0}})$ and the population size parameters (θs) are assigned diffuse gamma priors G(α,β) with shape parameter α=1 and with the mean of the distribution (α/β) matching the true value used in the simulation. Thus, for the low-mutation rate case, the priors were $\tau_{\text{0}}∼\text{G}(\text{1},\text{333})$, with mean 0.003 (3 differences per kb), and θ ∼G(1,500), with mean 0.002. For the high-mutation rate case, the priors are $\tau_{\text{0}}∼\text{G}(\text{1},\text{33})$ and θ ∼G(1,50). Note that the prior for divergence times at other nodes on the guide tree is generated from a uniform Dirichlet distribution (Yang and Rannala 2010). Each analysis was conducted twice, using rjMCMC algorithms 0 (with ϵ=2) and 1 (with α=2 and m=1) in BPP. The fine-tuning parameters ϵ in algorithm 0 and α and m in algorithm 1 are used to propose new parameters in the multispecies coalescent model (θ and τ) when a node on the guide tree is split (Yang and Rannala 2010, equations 3 and 6). The two runs or algorithms were used to check for consistency between runs. For each run, 20,000 samples were collected by sampling every two iterations after a burn-in of 5000 iterations. Samples from the two runs were then combined.

In summary, we considered two species trees (trees 1 and 2 in Fig. 1a), two sample sizes (with three or five sequences from each population), and two mutation rates (Table 1), with a total of eight parameter combinations. For each combination, 1000 replicate data sets were generated. Each replicate data set consisted of one mitochondrial locus and either one or five nuclear loci. Every locus consisted of either 24 or 40 sequences, with either three or five sequences from each of the eight populations. The data were then analyzed using two methods for inferring the guide tree: phylogenetic analysis of the mitochondrial locus using RAxML (and beast) and species-tree estimation from the nuclear loci using *beast. Those analyses are referred to later as RAxML + bpp (or beast + bpp if beast was used instead) and *beast + bpp. In the RAxML + bpp analysis, RAxML was used to analyze the mitochondrial locus to infer the guide tree, which was then used by bpp to analyze the nuclear data to delimit species. In the beast + bpp analysis, beast was used as an alternative to RAxML. In the *beast + bpp analysis, *beast was used to analyze the one or five nuclear loci to infer the guide tree, which is then used by bpp to analyze the same nuclear data to delimit species.

## Results

### Phylogenetic Errors in Guide-Tree Construction

We will focus on the small sample size, with three sequences sampled from each population, and discuss the results for the large sample size (with five sequences from each population) later. We first examine the phylogenetic errors in the guide-tree construction and then describe their impact on species delimitation.

**Figure 2.**
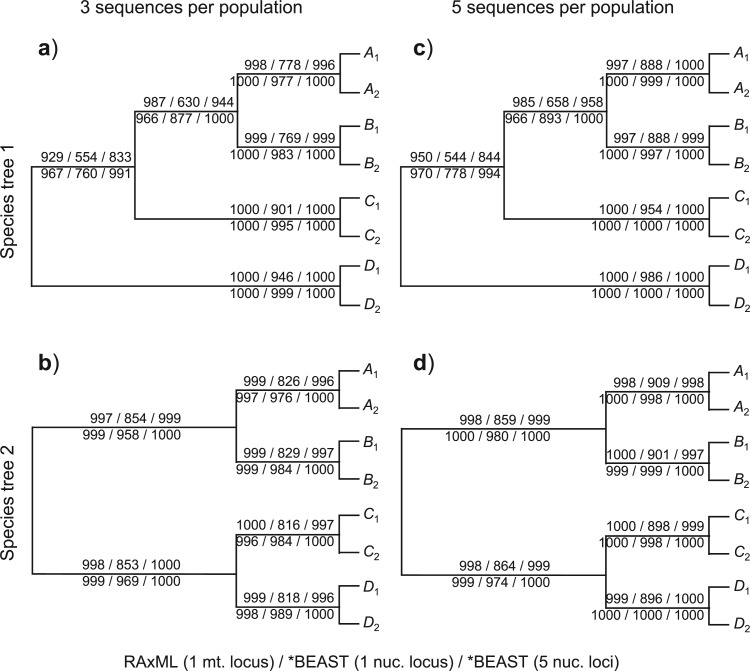
Frequency (out of 1000 replicates) at which each clade in the correct population (guide) tree is recovered by RAxML and *beast. The numbers above the branch are for the low-mutation rate while those below the branch are for the high rate. In each case, the three numbers are for RAxML for one mitochondrial locus, *beast with one nuclear locus, and *beast with five nuclear loci.

**Figure 3.**
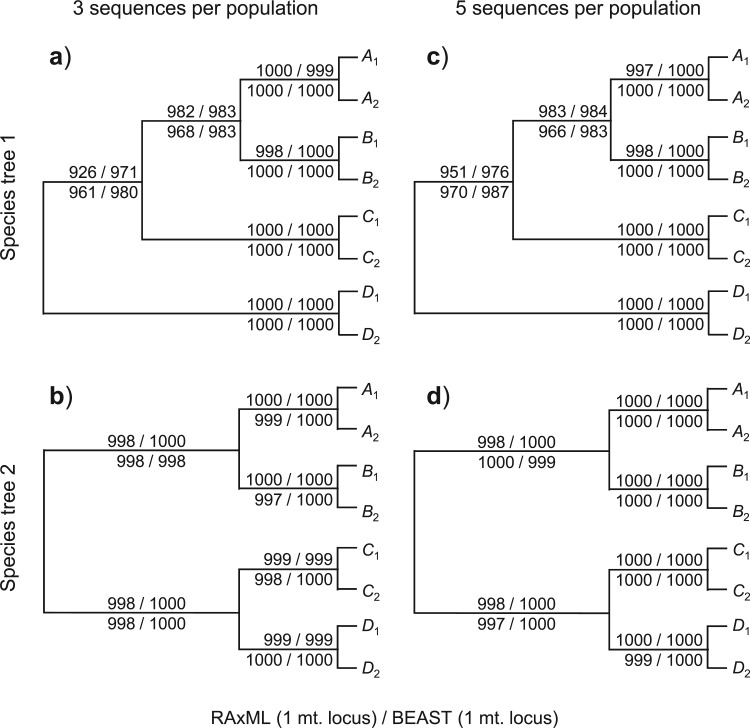
Frequency (out of 1000 replicates) at which each clade in the correct population (guide) tree is recovered by RAxML and beast in the analysis of the mitochondrial locus. The numbers above the branch are for the low-mutation rate whereas those below the branch are for the high rate. See legend to Figure 2.

Given the species trees of Figure 1a and our simulation design, the correct trees for the eight populations (i.e., the correct guide trees) are those shown in Figures 2 and 3. The proportion of replicates (out of 1000) in which each clade on the correct guide tree is recovered in the inferred guide tree is also shown (Figs. 2 and 3), calculated using the consense program in the phylip package version 3.69 (Felsenstein 2005). Note that we used only the population tree topology inferred by the two methods (RAxML/beast and *beast), and ignored any support measures for clades on the tree, such as the bootstrap support values calculated by RAxML and the posterior clade probabilities calculated by *beast. The results show clear effects of the species phylogeny (in particular, the lengths of the internal branches reflecting species divergence times), the mutation rate, and the number of loci. A longer internal branch on the species tree makes the concerned clade easier to recover. A higher mutation rate means that the sequences are more divergent and more informative about the phylogeny (Yang 1998). Similarly, more loci means more data so that the inference is more reliable. Those patterns are easy to understand and are similar to findings from numerous simulation studies that examine the performance of different phylogenetic methods (for review, see Yang [2006, Chapter 6]).

The RAxML analysis of the mitochondrial locus recovered almost all nodes with high probability, except the difficult clade *ABC* in tree 1, for which the probability is 93% and 96%, for the low- and high-mutation rates, respectively (Fig. 2a). We also conducted a Bayesian phylogenetic analysis of the same data using the program beast (with the same prior settings as for the single nuclear locus), with the results summarized in Figure 3. The probability of recovering the difficult clade *ABC* in tree 1 is 97% or 98% for the two mutation rates, which are slightly higher than for RAxML (93% and 96%) (Fig. 3a). The slightly poorer performance for RAxML may be due to the fact that the RAxML analysis assumed the more general GTR model with mid-point rooting, which may not be as efficient as the use of the JC69 model and molecular clock rooting (given that the data are simulated under JC69 and the clock). In general, both the ML and the Bayesian analysis of the mitochondrial locus recovered the true clades with very high probability (Figs. 2 and 3). Below we focus on the guide trees inferred using RAxML.

The *beast analysis of one nuclear locus performed poorly, especially at the low rate. For example, clade *ABC* in tree 1 is recovered in only 55% of replicate data sets at the low-mutation rate (Fig. 2a). A single locus at the low-mutation rate does not contain enough information to infer the correct guide tree. However, performance improved dramatically if the mutation rate was 10 times higher (with the probability of recovering clade *ABC* in tree 1 to be 76%, Fig. 2a) or if five loci were analyzed (with the probability of recovering clade *ABC* in tree 1 to be 83%, Fig. 2a). The four clades grouping the two populations of each species ($A_{\text{1}}A_{\text{2}},B_{\text{1}}B_{\text{2}},C_{\text{1}}C_{\text{2}}$, and $D_{\text{1}}D_{\text{2}})$ were recovered with high probabilities on both species trees by both methods except for the *beast analysis under the combination of a low rate and one nuclear locus.

### False-Positive Rate in Species Delimitation

In the species delimitation analysis by bpp, we considered a split of a node on the guide tree into different species to be well supported only if the posterior probability calculated by bpp was more than or equal to 95%. Thus, we defined the “false-positive rate” as the percentage of data replicates in which two populations of the same species ($A_{\text{1}}$ and $A_{\text{2}},B_{\text{1}}$ and $B_{\text{2}},C_{\text{1}}$ and $C_{\text{2}}$, or $D_{\text{1}}$ and $D_{\text{2}})$ are split into different species with posterior probability more than or equal to 95%. For example, if the true species tree is tree 2 of Figure 1a and the inferred guide tree is the tree on the right in Figure 1b, then we counted a false positive for splitting $A_{\text{1}}A_{\text{2}}$ if the posterior probability for splitting node 11 was more than or equal to 95%. The results are summarized in Tables 2 and 3 for species trees 1 and 2 of Figure 1a, respectively.

**Table 2.** Percentage of false positives splitting one species into two by bpp with posterior more than or equal to 95% in data simulated using tree 1, with three sequences sampled from each population

Nuclear Loci	Method	$A_{\text{1}}A_{\text{2}}$	$B_{\text{1}}B_{\text{2}}$	$C_{\text{1}}C_{\text{2}}$	$D_{\text{1}}D_{\text{2}}$
Low-mutation rate					
1 locus	RAxML	0.012	0.012	0.015	0.010
	*beast	0.083	0.083	0.047	0.037
5 loci	RAxML	0.009	0.004	0.004	0.004
	*beast	0.009	0.006	0.007	0.007
High-mutation rate					
1 locus	RAxML	0.034	0.042	0.035	0.026
	*beast	0.044	0.035	0.031	0.027
5 loci	RAxML	0.000	0.000	0.000	0.000
	*beast	0.000	0.000	0.000	0.000

Notes: Method refers to two methods for generating the guide tree: phylogenetic method applied to the mitochondrial locus (RAxML), and species-tree inference method applied to the nuclear loci (*beast).

**Table 3.** Percentage of false positives splitting one species into two by bpp with posterior more than or equal to 95% in data simulated using tree 2, with three sequences sampled from each population

Nuclear Loci	Method	$A_{\text{1}}A_{\text{2}}$	$B_{\text{1}}B_{\text{2}}$	$C_{\text{1}}C_{\text{2}}$	$D_{\text{1}}D_{\text{2}}$
Low-mutation rate					
1 locus	RAxML	0.007	0.009	0.003	0.011
	*beast	0.053	0.057	0.058	0.064
5 loci	RAxML	0.004	0.006	0.006	0.002
	*beast	0.006	0.005	0.009	0.006
High-mutation rate					
1 locus	RAxML	0.030	0.036	0.030	0.030
	*beast	0.043	0.037	0.040	0.036
5 loci	RAxML	0.001	0.001	0.002	0.002
	*beast	0.000	0.000	0.001	0.000

See note to Table 2.

The false-positive errors have contributions from two sources: errors in the inferred guide tree and errors in species delimitation by bpp. In the *beast + bpp analysis, the false-positive rate is much lower when five nuclear loci are used than when only one locus is used (Tables 2 and 3). For example, the error rate for splitting clades $A_{\text{1}}A_{\text{2}}$ and $B_{\text{1}}B_{\text{2}}$ on species tree 1 at the low-mutation rate was 8.3% for one nuclear locus and approximately 0.7% for five loci. This performance difference is due both to the improved accuracy of guide-tree inference (see Fig. 2) and to increased information content in the bpp analysis. In contrast, in the RAxML + bpp analysis, the performance improvement due to the increased number of nuclear loci is much less dramatic. For example, the error rate for splitting $A_{\text{1}}A_{\text{2}}$ and $B_{\text{1}}B_{\text{2}}$ on species tree 1 at the low-mutation rate was 1.2% for one nuclear locus and approximately 0.7% for five loci. In this analysis, there is no reduction in guide-tree estimation errors when more nuclear loci are used and the performance improvement is entirely due to the increased information content in the bpp analysis of the nuclear loci. Thus, errors in the guide-tree construction clearly contribute to false-positive errors in species delimitation by bpp.

**Figure 4.**
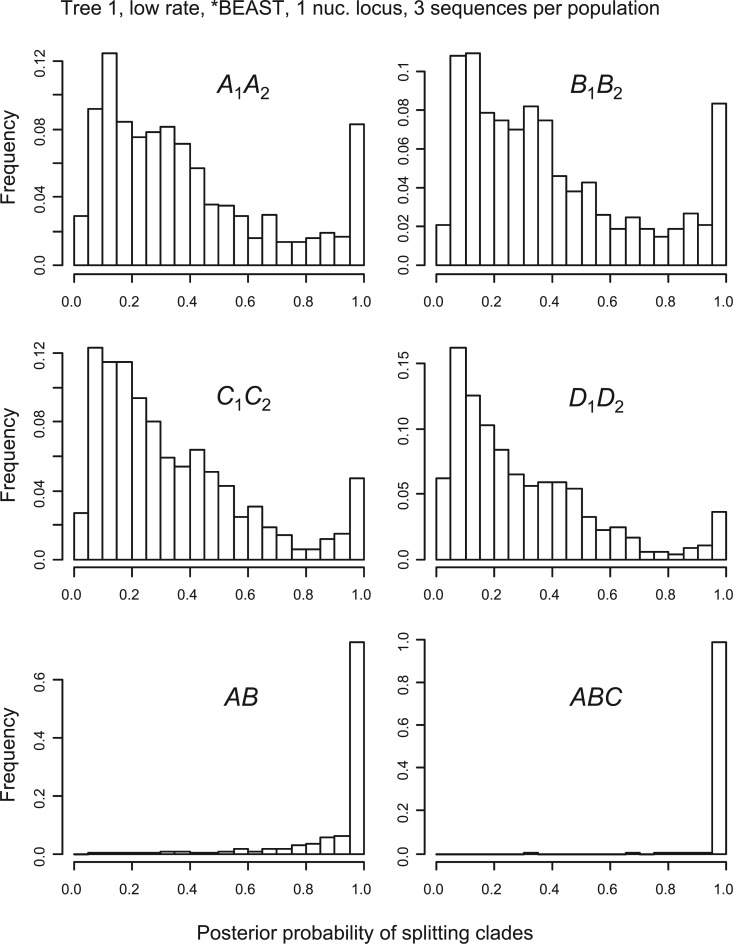
Histogram of posterior probabilities for splitting clades into different species by bpp in data of one locus, with three sequences sampled from each population at the locus, simulated using tree 1 at the low-mutation rate, when the guide tree was inferred using *beast. Each bin is of size 0.05. The frequencies in the last bin for splitting clades $A_{1}A_{2},B_{1}B_{2},C_{1}C_{2}$, and $D_{1}D_{2}$ are the false-positive rates listed in Table 2.

**Figure 5.**
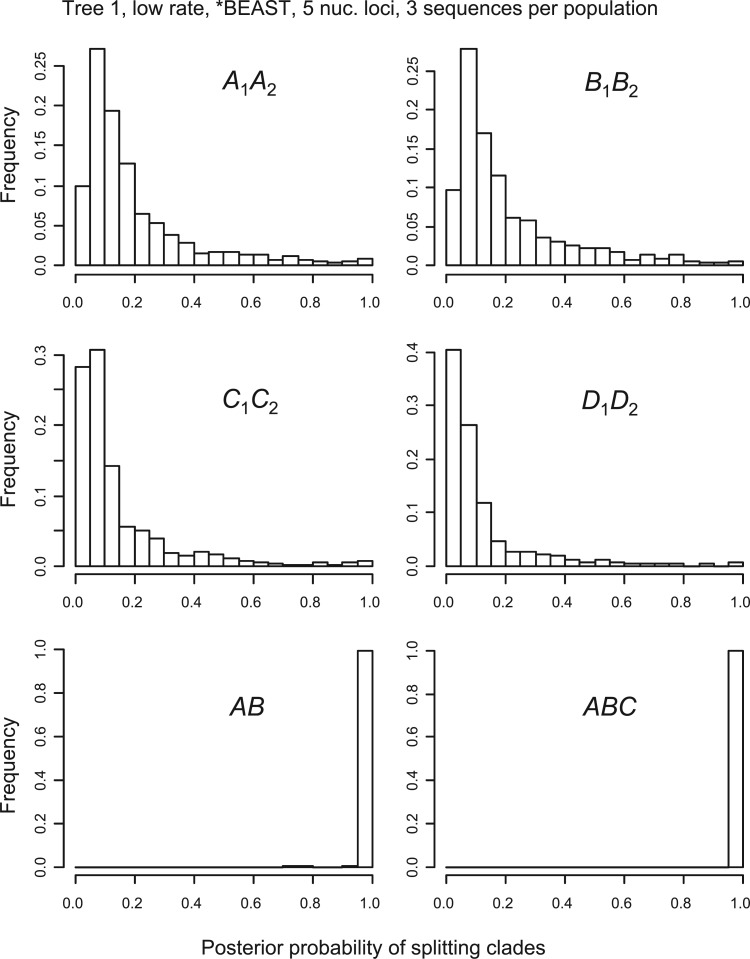
Histogram of posterior probabilities for splitting the clades by bpp in data of five loci, with three sequences per population at each locus, simulated using tree 1 with low-mutation rate, when the guide tree was inferred using *beast. See legend to Figure 4.

However, the false-positive rates in those simulations are overall quite low. In all cases except one, the false-positive rates were near or below the nominal rate of 5%. The exception is the case of *beast + bpp analysis of one nuclear locus at the low rate for species tree 1, in which bpp splits clades $A_{\text{1}}A_{\text{2}}$ and $B_{\text{1}}B_{\text{2}}$ in approximately 8% of replicates, slightly above the nominal 5%. In this case, phylogenetic errors in the guide tree inferred by *beast are very common, with clades $A_{\text{1}}A_{\text{2}}$ and $B_{\text{1}}B_{\text{2}}$ recovered in only 77% of the replicates (Fig. 2a). To understand why such high errors in the guide-tree inference did not lead to very high false positives in bpp species delimitation, we plot in Figures 4 and 5 the distributions (histograms) of posterior probabilities calculated by bpp (see also Tables 4 and 5 for the medians and quartiles, and online supplementary Figs. S1–S16 for other cases). With one locus (Fig. 4), the posterior probabilities for splitting clades $A_{\text{1}}A_{\text{2}}$ and $B_{\text{1}}B_{\text{2}}$ are spread-out. With five loci (Fig. 5), they shift towards 0 and become highly concentrated. Thus, in the data of a single nuclear locus, the posterior probabilities calculated by bpp did not often reach the 95% cut-off due to the lack of information. With more loci or at the higher mutation rate, the data become far more informative and the posterior probabilities become more extreme. However, in such cases, the guide tree tends to be correctly reconstructed (Fig. 2a) and bpp becomes increasingly accurate with lower rates of false positives and false negatives (Table 2).

**Table 4.** Median and quartiles (in parentheses) of posterior probabilities for splitting the specified clades by bpp in data simulated using tree 1, with three sequences per population

Nuclear Loci	Method	$A_{\text{1}}A_{\text{2}}$	$B_{\text{1}}B_{\text{2}}$	$C_{\text{1}}C_{\text{2}}$	$D_{\text{1}}D_{\text{2}}$	*AB*	*ABC*
Low-mutation rate							
1 locus	RAxML	0.315	0.308	0.269	0.223	0.990	1.000
		(0.171, 0.480)	(0.164, 0.484)	(0.120, 0.454)	(0.101, 0.407)	(0.915, 1.000)	(1.000, 1.000)
	*beast	0.310	0.322	0.262	0.228	0.996	1.000
		(0.153, 0.533)	(0.157, 0.556)	0.142, 0.467)	(0.109, 0.430)	(0.933, 1.000)	(1.000, 1.000)
5 loci	RAxML	0.135	0.135	0.080	0.061	1.000	1.000
		(0.077, 0.258)	(0.074, 0.258)	(0.046, 0.166)	(0.032, 0.132)	(1.000, 1.000)	(1.000, 1.000)
	*beast	0.130	0.137	0.082	0.063	1.000	1.000
		(0.077, 0.245)	(0.075, 0.270)	(0.046, 0.161)	(0.033, 0.131)	(1.000, 1.000)	(1.000, 1.000)
High-mutation rate							
1 locus	RAxML	0.042	0.046	0.024	0.018	1.000	1.000
		(0.021, 0.130)	(0.021, 0.150)	(0.011, 0.077)	(0.008, 0.060)	(0.995, 1.000)	(1.000, 1.000)
	*beast	0.039	0.039	0.025	0.019	1.000	1.000
		(0.019, 0.124)	(0.019, 0.112)	(0.011, 0.074)	(0.008, 0.065)	(0.993, 1.000)	(1.000, 1.000)
5 loci	RAxML	0.008	0.007	0.003	0.002	1.000	1.000
		(0.003, 0.017)	(0.003, 0.017)	(0.001, 0.009)	(0.000, 0.006)	(1.000, 1.000)	(1.000, 1.000)
	*beast	0.007	0.007	0.003	0.001	1.000	1.000
		(0.003, 0.016)	(0.003, 0.018)	(0.001, 0.009)	(0.000, 0.005)	(1.000, 1.000)	(1.000, 1.000)

Notes: The probability for splitting clade *ABCD* (the root) is 1 in every replicate data set.

**Table 5.** Median and quartiles (in parentheses) of posterior probabilities for splitting the specified clades by bpp in data simulated using tree 2, with three sequences per population

Nuclear Loci	Method	$A_{\text{1}}A_{\text{2}}$	$B_{\text{1}}B_{\text{2}}$	$C_{\text{1}}C_{\text{2}}$	$D_{\text{1}}D_{\text{2}}$	*AB*	*CD*
Low-mutation rate							
1 locus	RAxML	0.268	0.275	0.266	0.257	0.986	0.988
		(0.138, 0.427)	(0.144, 0.446)	(0.127, 0.418)	(0.140, 0.443)	(0.896, 0.999)	(0.916, 0.999)
	*beast	0.289	0.260	0.291	0.270	0.993	0.993
		(0.146, 0.504)	(0.136, 0.464)	(0.145, 0.503)	(0.136, 0.488)	(0.925, 1.000)	(0.916, 1.000)
5 loci	RAxML	0.106	0.107	0.110	0.109	1.000	1.000
		(0.062, 0.218)	(0.061, 0.215)	(0.062, 0.221)	(0.064, 0.207)	(1.000, 1.000)	(1.000, 1.000)
	*beast	0.113	0.110	0.112	0.107	1.000	1.000
		(0.062, 0.201)	(0.062, 0.219)	(0.063, 0.234)	(0.062, 0.204)	(1.000, 1.000)	(1.000, 1.000)
High-mutation rate							
1 locus	RAxML	0.034	0.040	0.036	0.037	1.000	1.000
		(0.016, 0.133)	(0.018, 0.113)	(0.017, 0.120)	(0.018, 0.118)	(0.992, 1.000)	(0.992, 1.000)
	*beast	0.036	0.032	0.040	0.038	1.000	1.000
		(0.018, 0.504)	(0.015, 0.464)	(0.018, 0.503)	(0.018, 0.488)	(0.990, 1.000)	(0.991, 1.000)
5 loci	RAxML	0.004	0.005	0.005	0.004	1.000	1.000
		(0.002, 0.012)	(0.002, 0.011)	(0.002, 0.012)	(0.002, 0.010)	(1.000, 1.000)	(1.000, 1.000)
	*beast	0.004	0.005	0.004	0.005	1.000	1.000
		(0.002, 0.010)	(0.002, 0.012)	(0.002, 0.011)	(0.002, 0.011)	(1.000, 1.000)	(1.000, 1.000)

Notes: The probability for splitting clade *ABCD* (the root) is 1 in every replicate data set.

The posterior probabilities for splitting clades *AB* and *ABC* on species tree 1 reflect the power of bpp to identify distinct species (Figs. 4 and 5 and Tables 4 and 5). Power is high even in the least informative data set of one nuclear locus at the low rate (Fig. 4), and is nearly 100% when five loci are analyzed (Fig. 5).

Note that the false-positive rate we calculate here is a Frequentist property, and there is no theory leading one to expect that the false-positive rate for a Bayesian method (bpp) will be less than 5%. In practice, however, many Bayesian methods are known to also have good Frequentist properties (e.g., Huelsenbeck and Rannala 2004). The bpp method of species delimitation appears to be one such method. Similarly, when the amount of data (e.g., the number of loci) or the amount of information in the data increases, the false-positive rates of bpp for splitting clades $A_{\text{1}}A_{\text{2}}$, $B_{\text{1}}B_{\text{2}},C_{\text{1}}C_{\text{2}}$, and $D_{\text{1}}D_{\text{2}}$ approach zero, rather than staying at the nominal 5% as in a likelihood ratio test. This is clearly seen from the dramatic reduction in the false-positive rates when the mutation rate was increased by 10-fold or when the number of loci was increased from 1 to 5 (Tables 2 and 3), and from the distribution of the posterior probabilities calculated by bpp for the four clades $A_{\text{1}}A_{\text{2}},B_{\text{1}}B_{\text{2}},C_{\text{1}}C_{\text{2}}$, and $D_{\text{1}}D_{\text{2}}$ (compare Figs. 4 with 5).

### The Impact of the Sample Size

We examined the effect of the sample size by increasing the number of sequences sampled from each population at each locus from 3 to 5, so that there are 40 sequences in the alignment at each locus. The probabilities with which the clades on the correct guide tree are recovered are shown in Figure 2c,d. The recovery probabilities are either very similar to or higher than the corresponding probabilities for the small sample size of Figure 2a,b. For example, in the RAxML analysis of the mitochondrial locus, the probability of recovering clade *ABC* in tree 1 is 95% and 97% for the low- and high-mutation rates, respectively, when five sequences per population are sampled (Fig. 2c), compared with 93% and 97% for the small sample size of three sequences per population (Fig. 2a). Note that for both the small and large sample sizes, a phylogeny of eight populations is inferred, so that the parameter space (and the number of parameters) of the inference problem remains unchanged even though the gene trees are larger. Thus, a larger sample means more data and more information.

The histograms of posterior probabilities for splitting clades on the correct guide tree for the large sample size are presented in online Supplementary Figures S17–S32. Compared with the corresponding results for the small sample size (Supplementary Figs. S1–S16), species delimitation by bpp performed in general better with the large sample size. For example, in the *beast + bpp analysis of one nuclear locus at the low-mutation rate (Supplementary Figs. S1 and S17), the posterior probabilities for splitting clades $A_{\text{1}}A_{\text{2}},B_{\text{1}}B_{\text{2}},C_{\text{1}}C_{\text{2}}$, and $D_{\text{1}}D_{\text{2}}$ (false positives) are lower in the large sample, indicating lower false positives, whereas the probability for splitting clade *AB* is higher, indicating higher power. The probability for splitting *ABC* is approximately 100% for both sample sizes. The better performance of bpp for the large sample size appears to be largely due to the increased information content for species delimitation since the improvement in guide-tree inference is moderate. A previous simulation found that increasing the number of sequences sampled from the same species improves species delimitation by bpp, leading to both reduction of false positives (over-splitting errors) and increase of power (correctly delimiting distinct species) (Zhang et al. 2011).

## Discussion

### Impact on Species Delimitation of Errors in the Estimated Guide Tree

We investigated the impact of possible errors in the guide tree on Bayesian species delimitation by bpp, using two approaches for constructing the guide tree: (i) phylogenetic analysis of a mitochondrial locus using ML and Bayesian methods (RAxML and beast) and (iii) species-tree estimation using independent nuclear loci (*beast). When the mutation rate was high, both approaches had a good chance of inferring the correct guide tree. When the mutation rate was low, the estimated guide trees might involve considerable errors, especially if only one nuclear locus was used. However, even in this case the false-positive rate in Bayesian species delimitation by bpp was not very high (the highest error rate being ∼8% when the nominal value is 5%). This is because when the sequence data lack information, the posterior probabilities calculated by bpp tend to be low and do not reach the 95% threshold. With more data, the posterior probabilities become more extreme, but in that case both guide-tree inference and species delimitation become highly accurate.

For multilocus nuclear data, one could conduct a phylogenetic analysis of the concatenated sequence alignment to generate a guide tree, using for example, RAxML. However, concatenation assumes that the same gene tree underlies all loci and fails to accommodate incomplete lineage sorting due to polymorphism in the ancestral species. We have not examined this alternative method since it is expected to be inferior to species-tree methods (such as *beast), which use the multispecies coalescent model to account for gene tree discordance across loci. For the mitochondrial locus, RAxML and beast perform similarly, but RAxML runs several orders of magnitude faster than beast. Our discussion has thus focused on RAxML analysis of the mitochondrial locus but we note that Bayesian programs such as beast and MrBayes are usable as well. We stress that our objective in this study is not to compare different phylogenetic reconstruction methods (such as RAxML and beast) but is instead to evaluate the impact of errors in estimated guide trees on the false-positive and false-negative errors in the downstream species delimitation analysis by bpp. In this regard, our results suggest that the false-positive errors are rather minor when the guide tree is generated using sampled sequence data. Our results complement rather than contradict the previous finding by Leaché and Fujita (2010) that bpp tends to over-split and generate excessive false positives if a random guide tree, which is most likely to be grossly wrong, is used. Users of bpp should take precautions against using grossly wrong guide trees for species delimitation analysis by bpp. If there are uncertainties concerning the phylogenetic relationships of the populations, the sensitivity of bpp analysis to the guide tree should be examined by using multiple guide trees derived using different strategies (as reviewed early). Furthermore, there is clearly a need to extend the algorithms in bpp to account properly for uncertainties in the guide tree.

### The Impact of Gene Flow

In our simulation, we assumed no gene flow (migration, hybridization, or introgression) after species divergence, and conflicts between gene trees from different genomic regions or between mitochondrial and nuclear loci are entirely due to ancestral polymorphism and incomplete lineage sorting. A previous simulation study has examined the impact of gene flow on Bayesian species delimitation by bpp (Zhang et al. 2011). It was found that small amounts of migration (with ≪1 expected immigrant per generation) had little impact on the performance of the method, whereas a single species was inferred if migration between populations was prevalent (say, with ≫1 immigrants per generation). In that study, gene flow was assumed to affect all loci uniformly and the guide tree was assumed to be correct. The effect of migration may be more difficult to predict if migration affects different parts of the genome differently, due to natural selection. For example, the pattern of gene flow may vary considerably across genome regions because some loci are responsible for species adaptations to different ecological habitats and are thus under strong selection whereas other loci are neutral and can cross species boundaries quite freely. As a result, incipient species may show “islands” of divergence between their genomes amidst a sea of gene flow (Ellegren et al. 2012; Martin et al. 2013). Discordance between mitochondrial and nuclear phylogenies may also result from such selective gene flow, which makes the use of the mitochondrial locus to construct the guide tree problematic.

### The Impact of Assignment Errors

In this study, we assumed that the population assignments were correct. In a recent simulation study, Olave et al. (2014) used structurama to assign individuals to populations and then used *beast to infer the guide tree, to evaluate the impact of errors in the upstream analysis (assignment and guide-tree construction) on the performance of bpp. They found that the error rates may be high when individuals are incorrectly assigned to populations, although bpp had excellent performance when assignment errors were absent. The results highlight the importance of reliable assignments to species delimitation by bpp. They also point to an interesting mismatch in the different steps of the delimitation process: although a few loci appeared to be sufficient for bpp to delimit species given the correct assignment, they were not enough for structurama to assign individuals to populations reliably. Nevertheless, a few issues with the design of the Olave et al. study make their results somewhat difficult to interpret. First, Olave et al. (2014; Fig. 2) used the number of inferred species to measure performance and failed to distinguish between the errors of over-splitting and under-splitting. Over-splitting appears to be a more serious error than under-splitting, as failure to delimit distinct species may simply be due to lack of power of the method or lack of information in the data. Second, Olave et al. (2014) used structurama to analyze the multilocus sequence data (treated as genotypes) to cluster the individuals into populations. The procedure mimics an unrealistic scenario in which multiple sympatric cryptic species exist in a sample with nothing to distinguish them *a priori*. Although the results suggest that a few loci of sequence data are insufficient for structurama to assign individuals to populations reliably, the impact of assignment errors on species delimitation by bpp under more realistic scenarios remains unknown. As discussed by Olave et al., traditional taxonomic boundaries, and morphological and geographical data may be available to determine the number of putative species and assign individuals to populations. Moreover, SNPs and microsatellites across multiple loci may be better suited than sequences for assigning individuals to populations.

## Supplementary Material

Supplementary Figures S1–S32: Distributions or histograms (out of 1000 replicate data sets) of posterior probabilities for splitting the clades on the correct guide tree, calculated by bpp. The 32 figures correspond to 32 simulation parameter settings, with two sample sizes (three or five sequences per population), two species trees (trees 1 or 2 in Fig. 1a), two mutation rates (Table 1), two guide-tree inference methods (RAxML: ML tree for the mitochondrial locus and *beast: species-tree inference from the nuclear loci), and two numbers of loci (one or five nuclear loci), with the last factor changing first. Thus, Supplementary Figures S1–S16 are for the small sample size of three sequences per population and Supplementary Figures S17–S32 are for the large sample size of five sequences per population. Supplementary Information related to this article has been deposited at Dryad under http://dx.doi.org/10.5061/dryad.m1r32.

## Funding

This study is supported by a grant from the Biotechnological and Biological Sciences Research Council (BBSRC) and a Royal Society-Wolfson Merit Award, both to Z.Y. C.Z. wishes to acknowledge UCL CoMPLEX for a Visiting Researcher Award, which allowed him to visit London.
